# Genome-Wide Identification and Characterization of the Calmodulin-Binding Transcription Activators (CAMTA) Gene Family in *Brassica* U-Triangle Species and Its Potential Role in Response to Phytohormones and Abiotic Stresses

**DOI:** 10.3390/plants15030480

**Published:** 2026-02-03

**Authors:** Qinghui Wang, Si Chen, Haobo Li, Pan Niu, Xinyuan Wang, Huiyan Zhao, Huafang Wan, Cunmin Qu, Daixiang Xu

**Affiliations:** Integrative Science Center of Germplasm Creation in Western China (CHONGQING) Science City, College of Agronomy and Biotechnology, Southwest University, Chongqing 400715, China; cherylw@email.swu.edu.cn (Q.W.); cs19960301@email.swu.edu.cn (S.C.); sanji2022@email.swu.edu.cn (H.L.); m18234893220@163.com (P.N.); w2840680935@email.swu.edu.cn (X.W.); zhaohuiyan@swu.edu.cn (H.Z.); wanhua05@swu.edu.cn (H.W.)

**Keywords:** calmodulin-binding transcription activators (CAMTAs), U-triangle species, *Brassica napus*, gene expression, abiotic stress, phytohormones

## Abstract

Calmodulin-binding transcription activators (CAMTAs) are pivotal regulators decoding calcium signals, with crucial roles in plant development, hormone responses, and adaptation to abiotic stresses. Although extensive research has been conducted on CAMTAs in model plants such as *Arabidopsis thaliana*, a comprehensive genome-wide analysis of the CAMTA gene family across the economically important *Brassica* U-triangle species has not been performed. In this study, we systematically identified and characterized 64 CAMTA genes from the genomes of *Brassica* U-triangle species. Phylogenetic analysis classified these genes into four conserved groups, a finding corroborated by analyses of gene structure and conserved motifs. These analyses revealed strong evolutionary preservation of functional domains, especially the calmodulin-binding domain (CaMBD). Chromosomal distribution and collinearity assessment highlighted the significant impact of polyploidization on the expansion of the CAMTA family, with most orthologous pairs being under purifying selection. *Cis*-element analysis in promoters uncovered an abundance of stress- and hormone-related elements, suggesting diverse regulatory roles for these genes. Furthermore, RNA-Seq and RT-qPCR expression profiling demonstrated that *BnaCAMTA* genes exhibit tissue-specific expression and are dynamically responsive to various phytohormones (ABA, JA, and GA) and abiotic stresses (salt and drought), particularly in the root. Notably, *BnaCAMTA5.2*, which was prioritized among several validated candidates, mediates the antagonistic regulation of hypocotyl and root growth under GA and salt stress, indicating its key role in balancing growth promotion and stress adaptation. Additionally, we identified a set of stress-related miRNAs that potentially target *BnaCAMTAs*, suggesting a potential layer of post-transcriptional regulation. Our results provide valuable insights into the evolutionary and functional diversity of CAMTA genes in *Brassica* U-triangle species and lay a foundation for further research into their roles in enhancing stress resistance in *B. napus*.

## 1. Introduction

The genus *Brassica* comprises a diverse array of economically and nutritionally significant vegetable and oilseed crops [1]. Among these, the U-triangle represents a classic and central evolutionary model that clearly delineates the genetic relationships among six major species within the genus. This model describes how three diploid progenitor species—*B. rapa* (AA), *B. nigra* (BB), and *B. oleracea* (CC)—underwent pairwise hybridization and genome doubling, giving rise to three allopolyploid species: *B. juncea* (AABB), *B. napus* (AACC), and *B. carinata* (BBCC) [2]. This polyploidization process has driven significant gene family expansion and genomic innovation within the genus. The completion of chromosome-scale genome assemblies for all six species provides vital genomic resources for further research [3,4,5,6,7,8].

*B. napus* is the third largest oil crop globally after oil palm and soybean and serves as a crucial source of both edible oil and biofuel [9,10]. Although *B. napus* is moderately salt-tolerant [11], saline–alkali soils still pose a significant challenge to its cultivation. In China, saline–alkali soils account for 5.01% of the total land area, representing a substantial land resource for the expansion of *B. napus* cultivation [12]. Moreover, other abiotic stresses, such as drought, also pose significant threats to the productivity of *B. napus* [13]. The allopolyploid species within the *Brassica* U-triangle have demonstrably evolved superior tolerance to abiotic stresses, including salinity, compared to their diploid progenitors [14]. Capitalizing on the genetic diversity to improve stress resilience in *B. napus* represents a pivotal breeding strategy. However, successful implementation critically depends on elucidating the key molecular regulators that confer such tolerance. Among the various signaling cascades implicated, calcium (Ca^2+^) signaling serves as a ubiquitous secondary messenger and central hub for initiating plant adaptive responses to abiotic stresses [15].

Calcium ion signals are transmitted and decoded by a diverse array of calcium sensor proteins, including calmodulins (CaMs), calmodulin-like proteins (CMLs), calcium-dependent protein kinases (CDPKs), and calcineurin B-like proteins (CBLs) [16,17,18]. These sensors enable plants to respond to various stimuli by mediating increases in cytosolic calcium concentration ([Ca^2+^]cyt) [19]. CaM is involved in regulating multiple cellular processes, such as stress responses and plant development [20]. Upon binding calcium ions, CaM can modulate the activity of specific transcription factors, such as calmodulin-binding transcription activators (CAMTAs) [21]. The activity of CAMTA is modulated through the binding of CaM to its conserved calmodulin-binding domain (CaMBD) upon Ca^2+^ activation, thereby regulating its transcriptional function [22].

CAMTAs, known as signal-responsive proteins, were initially identified during the screening of CaM-binding proteins in tobacco [23]. Structurally, CAMTA proteins contain multiple conserved domains arranged sequentially from the N- to the C-terminus, including a distinctive CG-1 DNA-binding domain that includes a nuclear localization signal (NLS), a transcription-associated Immunoglobulin-like (TIG) domain, which facilitates non-specific DNA binding, Ankyrin (ANK) repeats that mediate protein–protein interactions, an IQ motif (IQXXXRGXXXR), and a Ca^2+^-dependent calmodulin (CaM)-binding domain [24,25,26,27].

The CAMTA transcription factors play multifaceted roles in plant development and stress responses. In *Arabidopsis* thaliana, CAMTAs are involved in the JA (jasmonic acid) signaling pathway [28] and regulate both IAA (indole-3-acetic acid) and ABA (abscisic acid) responses [22,29]. CAMTAs are also central to abiotic stress tolerance in *Arabidopsis*, with *AtCAMTA1* enhancing general stress resistance [30] and other family members contributing to cold stress [29]. Beyond *Arabidopsis*, CAMTA homologs function in hormone signaling in other species, such as in the JA pathways of *Camellia sinensis* and *Prunus persica* [31,32], the IAA response in *Durio zibethinus* [33], and the ABA response in *Rosa chinensis* [34]. These findings highlight the conserved roles of CAMTAs across different plant species. Additionally, CAMTA orthologs in other species also play crucial roles in abiotic stress responses. *GmCAMTA12* from *Glycine max* [35], *HmCAMTA2* from *Heimia myrtifolia* [36], and *DzCAMTA3* from *D. zibethinus* [33] enhance general stress tolerance. CAMTAs contribute to cold stress resistance in *Salvia miltiorrhiza* and *C. sinensis* [31,37], show a positive response to heavy metal stress in *Solanum lycopersicum* [19], and improve salt tolerance across multiple species [38,39,40,41]. Thus, research in *Arabidopsis* provides a foundation for understanding the conserved and diverse functions of the CAMTA family.

In this study, we identified 64 CAMTA genes in the genomes of *Brassica* U-triangle species and analyzed their sequence characteristics, chromosomal distribution, and phylogenetic relationships. Expression patterns of *BnaCAMTAs* under phytohormones and abiotic stresses were analyzed to uncover their regulatory roles in these responses. Furthermore, we selected multiple candidate genes, including *BnaCAMTA5.2*, for experimental validation to investigate their potential roles in mediating the antagonistic interaction between GA signaling and salt stress, aiming to elucidate the mechanism by which CAMTAs coordinate growth and stress responses. These findings provide an important foundation for further understanding the expression regulation of CAMTA in *B. napus* in response to hormones and abiotic stress and offer a theoretical basis for breeding stress-resistant *B. napus* varieties.

## 2. Results

### 2.1. Identification of CAMTA Family Genes in Brassica U-Triangle Species

Using the protein sequence of *AtCAMTAs* as a query for a BLASTP search, we identified 64 CAMTA family members in *Brassica* U-triangle species. In detail, we identified 10 in *B. carinata*, 5 in *B. oleracea*, 14 in *B. juncea*, 18 in *B. napus*, 8 in *B. nigra*, and 9 in *B. rapa* (Table S1). These genes were designated as *CAMTA1.1* to *CAMTA6.2* based on their sequence similarity with *AtCAMTAs* and their chromosomal locations. Notably, *CAMTA2* was identified in *B. napus*, but no homolog was found in its diploid ancestor, *B. oleracea*, suggesting gene duplication or de novo gene formation. In contrast, a functional homolog was present in *B. rapa*, the other progenitor. Following interspecific hybridization and genome doubling between *B. oleracea* and *B. rapa* to form allotetraploid *B. napus*, this functional *CAMTA2* from *B. rapa* was introduced and retained in the *B. napus* genome.

The CAMTA proteins in U-triangle species ranged from 845 to 1396 amino acids in length, with molecular weights (MWs) ranging from 96.18 to 157.09 kDa. *BraCAMTA6* had the highest isoelectric point (pI = 7.63), while *BraCAMTA1* had the lowest isoelectric point (pI = 5.1). Most CAMTA proteins showed instability indices over 40, indicating general instability, except for *BjuCAMTA4.4* and *BcaCAMTA4.2*. Subcellular localization prediction results suggested that all CAMTA family members were localized in the nucleus, consistent with their roles as transcription factors.

### 2.2. Phylogenetic Analysis of CAMTA Proteins in the Brassica U-Triangle Species

**Figure 1:**
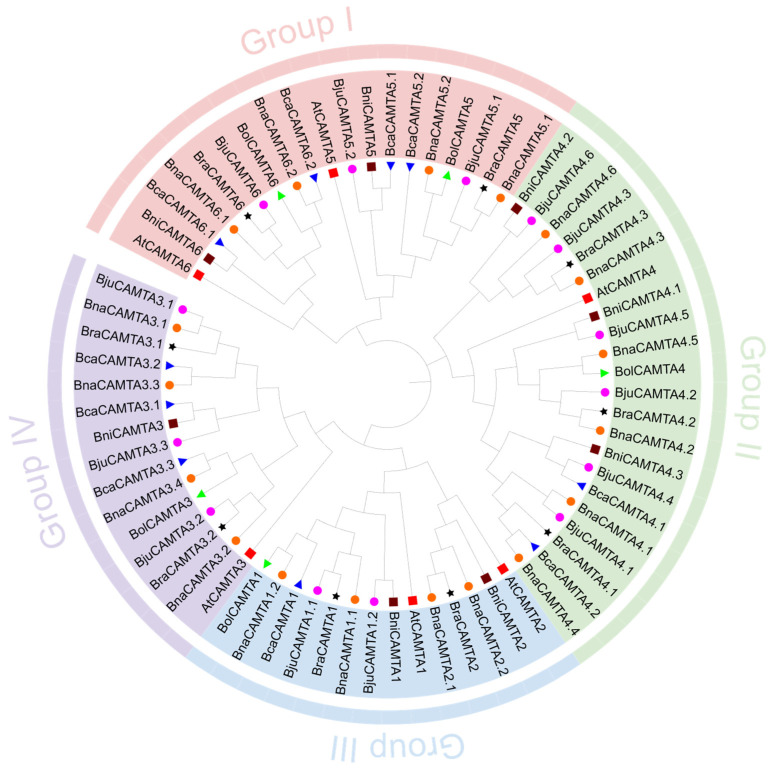
Phylogenetic tree of the CAMTA protein family from *A. thaliana* and *Brassica* U-triangle. Four groups (I–IV) are represented in pink, green, blue, and purple, respectively. The species *A. thaliana* (At), *B. carinata* (Bca), *B. oleracea* (Bol), *B. juncea* (Bju), *B. napus* (Bna), *B. nigra* (Bni), and *B. rapa* (Bra) are denoted by a red square, a blue triangle, a green triangle, a pink circle, an orange circle, a brown square, and a black star, respectively.

A phylogenetic tree was constructed using 70 CAMTA protein sequences from *A. thaliana* and *Brassica* U-triangle species to explore their evolutionary relationships. Based on their clustering relationships with *AtCAMTAs*, all proteins were classified into four distinct groups, designated as I to IV (Figure 1). Notably, Group III and Group IV were situated within the same major phylogenetic branch. Each group comprised representatives from all seven species, and the CAMTAs from the same *A. thaliana* gene exhibited closer relationships, indicative of a high degree of evolutionary conservation. Group II harbored the largest number of members, including four *AtCAMTAs*, two *BcaCAMTAs*, one *BolCAMTA*, six *BjuCAMTAs*, six *BnaCAMTAs*, three *BniCAMTAs*, and three *BraCAMTAs*. Group III contained the fewest homologous genes, only including *AtCAMTA1* and *AtCAMTA2*, plus one *BcaCAMTA*, one *BolCAMTA*, two *BjuCAMTAs*, four *BnaCAMTAs*, two *BniCAMTAs*, and two *BraCAMTAs*. Group I contained 19 proteins, and Group IV encompassed 15.

### 2.3. Multiple Sequence Alignment of CAMTA Proteins in Brassica U-Triangle Species

**Figure 2:**
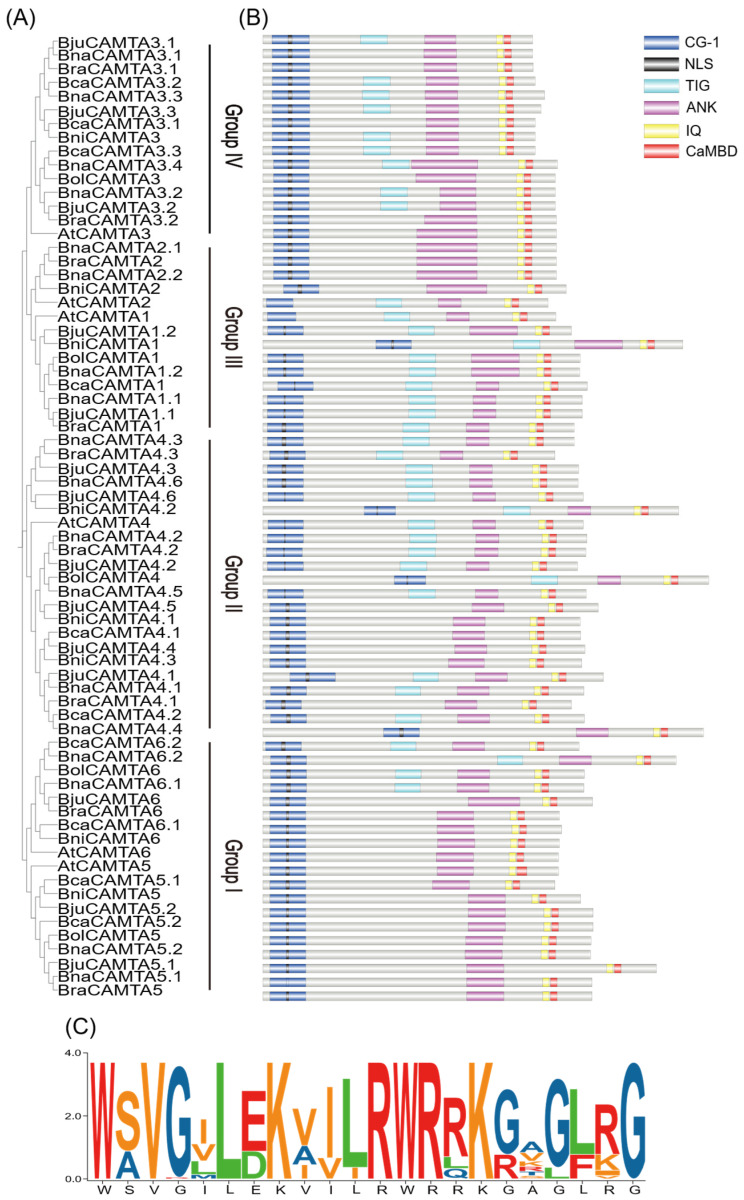
Sequence analysis of CAMTA family members in *Arabidopsis* and the *Brassica* U-triangle species. (**A**) Phylogenetic tree of 70 CAMTAs (identical to Figure 1); (**B**) Schematic representation of domains of CAMTA proteins. (**C**) WebLogo analysis of the conservation in the CaMBD domain.

The phylogenetic analysis revealed that the canonical CAMTA domain architecture is largely conserved across the four groups (Figure 2B). However, some variability was also observed, such as the loss of the TIG domain, which occurred sporadically across multiple lineages. These divergent forms were already present in the diploid progenitors and were subsequently inherited by the allotetraploids, suggesting they represent stable evolutionary variants rather than random anomalies. This variability may potentially contribute to functional diversification within the CAMTA gene family (Figure 2B). One key domain within CAMTA proteins, the CaMBD domain, was shown to be critically important for their biological functions [31]. To further investigate its conservation, sequence alignments were performed on the regions from *Arabidopsis* and *Brassica* U-triangle species. The analysis revealed a conserved functional motif within the CaMBD domain, namely WSVGILEKVILRWRRKGAGLRG (Figure 2C), which is consistent with the previously reported consensus [19]. This result further corroborates the evolutionary stability of the CaMBD domain, implying that its functional residues have remained highly conserved to maintain core biological activities.

### 2.4. Conserved Motifs and Gene Structures of CAMTA Family Members in Brassica U-Triangle Species

**Figure 3:**
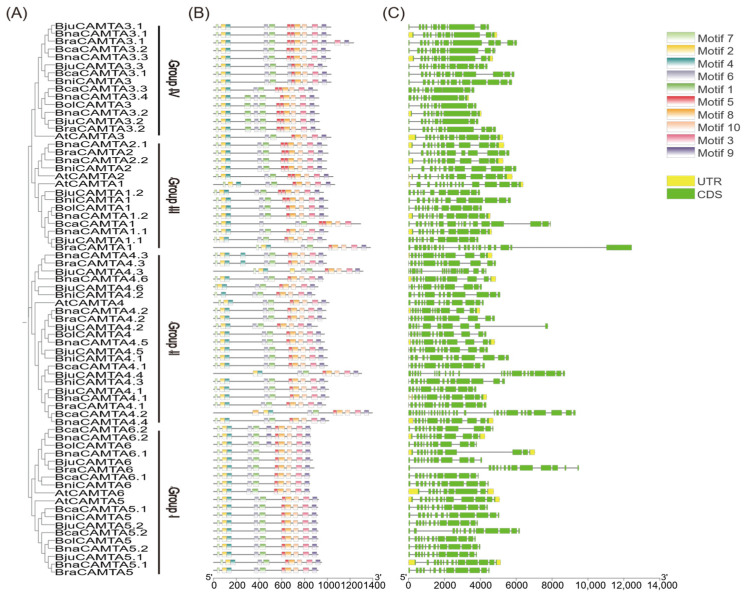
Phylogenetic tree, motif distributions, and gene structure analysis of CAMTAs in *Arabidopsis* and the *Brassica* U-triangle species. (**A**) Phylogenetic tree of 70 CAMTAs (identical to Figure 1). (**B**) Conserved motifs of CAMTA proteins. The schematic depicts ten motif instances, each represented by a colored box. The black lines indicate relative lengths of the proteins. (**C**) Gene structure of the CAMTA family. Coding sequences (CDS) are represented by green boxes, introns by gray lines, and untranslated regions (UTRs) by yellow boxes.

In conjunction with the evolutionary tree (Figure 3A), an analysis of conserved motifs was performed, revealing a total of 10 motifs across the 70 CAMTA proteins (Figure 3B). Most CAMTA proteins contained these 10 motifs. However, *BniCAMTA4.2* and *BniCAMTA4.3* were exceptions, as they lacked Motif 2 and Motif 7, respectively (Figure 3B). Proteins within the same groups shared nearly identical motif compositions and sequential arrangements. For instance, most Group IV members possessed two copies of Motif 5, highlighting the strong evolutionary conservation of this motif. Importantly, Motif 2, Motif 4, and Motif 7 constitute the CG-1 domain, a feature universally present in all CAMTA proteins.

Gene structure analysis revealed substantial divergence in exon number across the CAMTA family, with variations ranging from 9 (*BnaCAMTA3.4*) to 29 (*BcaCAMTA4.2*) (Figure 3C). This extensive structural diversity likely reflects genomic plasticity, possibly driven by mechanisms such as intron gain/loss or sliding. Notably, the variation in exon–intron structures followed a non-random pattern, showing strong correlation with phylogenetic groupings. Despite overall divergence, high structural similarity was maintained among members of the same groups. For instance, homologous genes in diploid ancestors and their allopolyploid descendants exhibited conserved structural features. This indicates that the gene structures of major CAMTA lineages were established prior to polyploidization and have been preserved under evolutionary constraints. The strong correlation between gene structure and phylogeny validates their established evolutionary relationships. Furthermore, the observed structural variations among different clades suggest potential functional divergence.

### 2.5. Chromosomal Distribution of CAMTA Genes in Brassica U-Triangle Species

**Figure 4:**
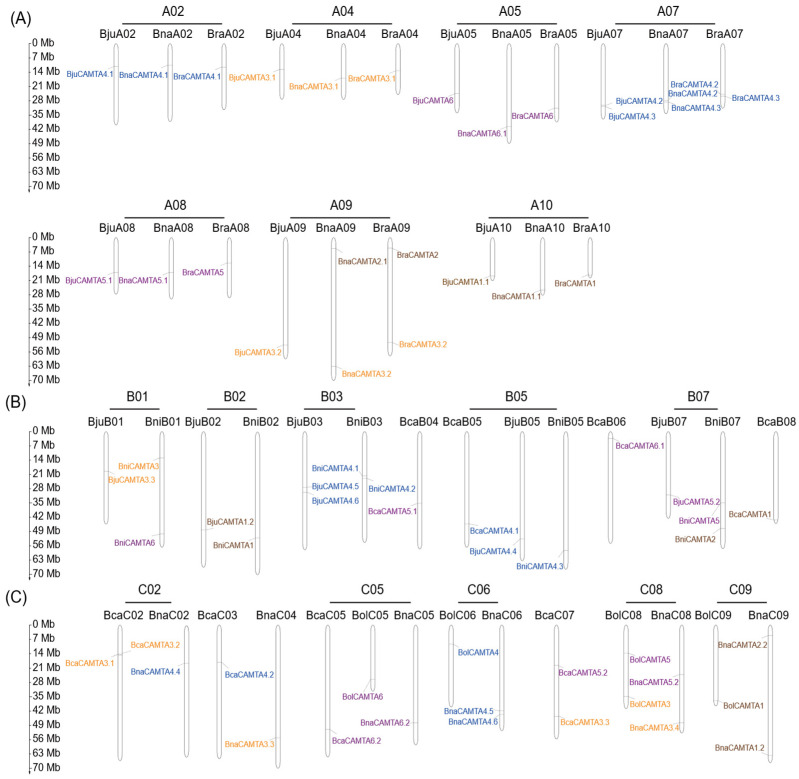
Chromosome distribution of CAMTA genes in *Brassica* U-triangle species. (**A**) Chromosomal distribution of CAMTA genes in the A subgenomes in *B. juncea*, *B. napus*, and *B. rapa*; (**B**) Chromosomal distribution of CAMTA genes in the B subgenomes in *B. juncea*, *B. nigra*, and *B. carinata*; (**C**) Chromosomal distribution of CAMTA genes in the C subgenomes in *B. napus*, *B. carinata*, and *B. oleracea*. The scale on the left indicates the physical length (in megabases, Mb) of the chromosomes shown. Genes in the same groups are shown in the same colors, with Group I in purple, Group II in blue, Group III in brown, and Group IV in orange.

The 64 CAMTA genes were mapped across 49 chromosomes of U-triangle species, exhibiting a non-random and uneven distribution (Figure 4). Specifically, the A subgenome (26 genes), B subgenome (18 genes), and C subgenome (20 genes) showed distinct patterns. In *B. rapa* (AA), CAMTA genes were identified on chromosomes A02, A04, A05, A07, A08, A09, and A10. *B. nigra* (BB) had CAMTA genes on chromosomes B01, B02, B03, B05, and B07. *B. oleracea* (CC) contained CAMTA genes on chromosomes C05, C06, C08, and C09. The allotetraploid species *B. juncea* (AABB) harbored CAMTA genes on both the A subgenome (chromosomes A02, A04, A05, A07, A08, A09, and A10) and the B subgenome (chromosomes B01, B02, B03, B05, and B07). *B. napus* (AACC) had CAMTA genes on the A subgenome (chromosomes A02, A04, A05, A07, A08, A09, and A10) and the C subgenome (chromosomes C02, C04, C05, C06, C08, and C09). *B. carinata* (BBCC) possessed CAMTA genes on the B subgenome (chromosomes B04, B05, B06, and B08) and the C subgenome (chromosomes C02, C03, C05, C07, and C09). Notably, the number of CAMTA genes was consistent between *B. rapa*, *B. juncea*, and *B. napus* on the A subgenome, and between *B. nigra* and *B. juncea* on the B subgenome, except for those on the BniB01 subgenome. Additionally, CAMTA genes belonging to the same groups were often located in parallel positions, indicating extensive collinearity.

The distribution of the 64 CAMTA genes across the 49 chromosomes of *Brassica* U-triangle species revealed pronounced clustering on specific chromosomes, such as A09 and C08, and on certain chromosomal arms. This clustering suggests the presence of genomic hotspots that favor gene retention or insertion. The different numbers of CAMTA genes among the A, B, and C subgenomes reflect divergent evolutionary trajectories, such as unequal gene loss or duplication, following polyploidization. A strong macrosyntenic relationship was maintained between the diploid progenitors and the corresponding subgenomes of the derived allopolyploids. For example, the conservation of CAMTA gene locations on chromosome A09 from *B. rapa* to the homologous chromosome in *B. napus* highlights the remarkable stability in genomic structure after hybridization and genome doubling. Furthermore, members of the same groups (I–IV) frequently occupied comparable chromosomal positions across different species, as indicated by the same colors in Figure 4. This spatial consistency provides physical evidence supporting the evolutionary relationships deduced from the phylogenetic tree, effectively linking sequence-based homology with chromosomal synteny.

### 2.6. Collinearity Analysis of CAMTA Genes in Brassica U-Triangle Species

**Figure 5:**
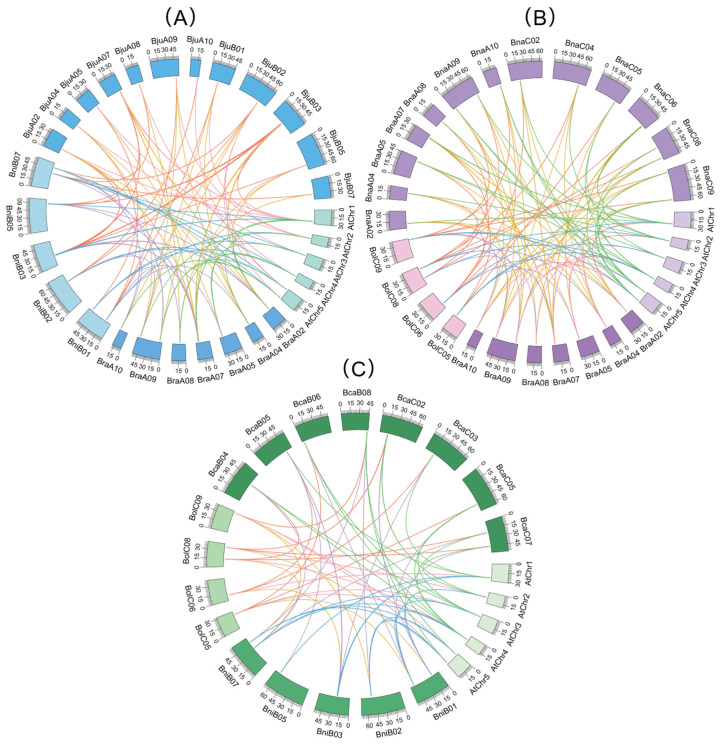
Collinearity analysis of CAMTA family genes between *A. thaliana* and the U-triangle species. (**A**) CAMTAs collinearity analysis in Group A among *A. thaliana*, *B. rapa*, *B. nigra*, and *B. juncea*; (**B**) CAMTAs collinearity analysis in Group B among *A. thaliana*, *B. rapa*, *B. oleracea*, and *B. napus*; (**C**) CAMTAs collinearity analysis in Group C among *A. thaliana*, *B. oleracea*, *B. nigra*, and *B. carinata*. Connecting lines are color-coded to represent distinct syntenic relationships. The scales represent the length of the chromosomes.

Orthologous gene pairs were identified after classifying the species into three evolutionary groups, each comprising *A. thaliana*, an allopolyploid species, and its two diploid progenitors (Figure 5). Group A (Figure 5A) comprised *A. thaliana* and *B. rapa* (11 pairs of orthologous genes), *A. thaliana* and *B. nigra* (12 pairs), *A. thaliana* and *B. juncea* (14 pairs), *B. nigra* and *B. juncea* (24 pairs), and *B. juncea* and *B. rapa* (27 pairs). Group B (Figure 5B) contained *A. thaliana* and *B. oleracea* (7 pairs), *A. thaliana* and *B. napus* (24 pairs), *A. thaliana* and *B. rapa* (11 pairs), *B. oleracea* and *B. napus* (16 pairs), and *B. napus* and *B. rapa* (38 pairs). Group C (Figure 5C) consisted of *A. thaliana* and *B. nigra* (12 pairs), *A. thaliana* and *B. carinata* (14 pairs), *A. thaliana* and *B. oleracea* (7 pairs), *B. nigra* and *B. carinata* (13 pairs), and *B. carinata* and *B. oleracea* (10 pairs). These findings demonstrated that collinear CAMTA homologs exhibit widespread genomic distribution with strong evolutionary conservation. This conservation follows three distinct evolutionary trajectories: from *A. thaliana* to the diploid *Brassica* species, from *A. thaliana* to the allotetraploids, and from the diploid ancestors to their respective allotetraploid descendants within the U-triangle species. To infer evolutionary constraints, we calculated the ratios of nonsynonymous (*Ka*) to synonymous substitutions (*Ks*) rates for the orthologous CAMTA gene pairs (Table S2). The *Ka/Ks* ratios ranged from 0.02 to 0.78, indicating that CAMTA family genes in the seven species might have likely undergone strong purifying selection following the duplication events that gave rise to the allotetraploid species of the U-triangle. This suggests that the functions of CAMTA genes have been strictly conserved following polyploidization events.

### 2.7. Cis-Element Analysis in the Promoter Regions of CAMTAs in Brassica U-Triangle Species

**Figure 6:**
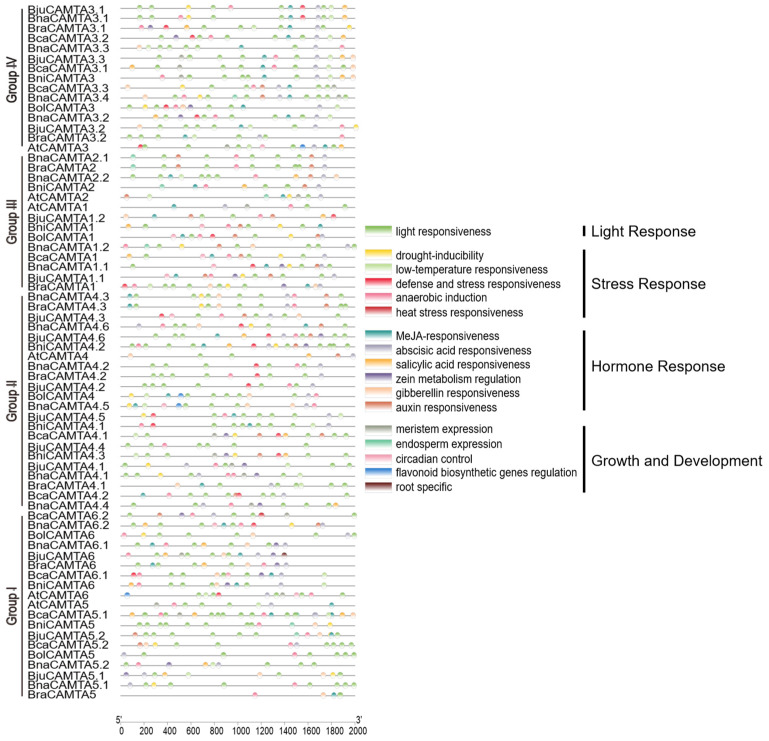
*Cis*-element prediction in CAMTA promoter regions of *A. thaliana* and U-triangle species. The scale at the bottom indicates the length of the sequence. The *cis*-elements are indicated by different colors.

To elucidate the potential regulatory mechanisms underlying CAMTA gene expression, a 2000 bp region upstream of the transcription start site was extracted for the *cis*-element prediction. The analysis of *cis*-elements in CAMTA promoters revealed distinct patterns across species (Figure 6 and Table S4 for a complete summary). At the species level, the allotetraploids *B. napus*, *B. juncea*, and *B. carinata* exhibited a greater abundance and diversity of stress- and hormone-responsive elements compared to their diploid progenitors. This pattern is not merely the sum of the two ancestral genomes but likely also results from genomic expansion following allopolyploidization. In *B. napus*, *BnaCAMTA4.5* contained the most diverse types of *cis*-elements (9 types), while *BnaCAMTA4.2* and *BnaCAMTA5.1* contained the fewest (3 types). Furthermore, all *BnaCAMTA* promoters contained phytohormone- and stress-responsive elements. Notably, light-responsive elements were ubiquitously present in all promoters, underscoring their fundamental role in CAMTA regulation. These patterns indicate that CAMTA regulation is shaped by evolutionary lineage.

### 2.8. Spatiotemporal Expression Profiles of BnaCAMTAs

To elucidate the expression patterns of *BnaCAMTA* genes across various organs and developmental stages, we analyzed the transcriptome data (with expression levels quantified as Fragments Per Kilobase of transcript per Million mapped reads, FPKM) from the *B. napus* cultivar Zhongshuang 11 (ZS11), retrieved from the BnIR database (https://yanglab.hzau.edu.cn/BnIR/expression_zs11; accessed on 3 September 2025). The results revealed distinct group-specific expression patterns of *BnaCAMTA* genes (Figure S1). The highest expression levels of most CAMTA genes in Group IV were detected in roots and siliques, with notable exceptions of *BnaCAMTA3.2* and *BnaCAMTA3.4*, which were expressed at lower levels. In contrast, members in Group I generally displayed the highest transcript levels, particularly in siliques and leaves, suggesting a prominent role in these organs. The expression profiles of CAMTA genes in Groups II and III were more specialized, with elevated levels in stems and developing siliques. While most CAMTA genes were expressed across all stages examined, significant transcriptional changes were observed during silique development. These findings collectively indicate that CAMTA homologs in *B. napus* have undergone functional diversification. Each group exhibits distinct spatial or temporal expression preferences, potentially reflecting their specific roles in growth and development.

### 2.9. Expression Profiles of BnaCAMTAs Under Phytohormone Treatments and Abiotic Stresses

**Figure 7:**
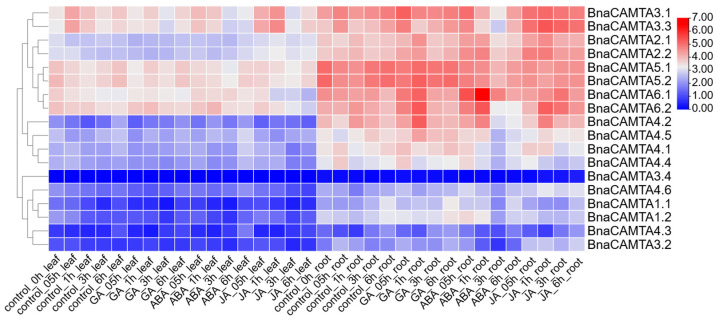
Heatmap analysis of *BnaCAMTAs* expression profiles in leaves and roots of ZS11 under phytohormone treatments; the relative expression of the *BnaCAMTAs* is represented as Log_2_ (FPKM value + 1). Labels 0.5 h, 1 h, 3 h, and 6 h represent hours after treatment.

To investigate the expression profiles of 18 *BnaCAMTA* genes from ZS11 in response to phytohormone or stress treatments, we utilized the published RNA-Seq data (https://yanglab.hzau.edu.cn/BnIR/expression_zs11; accessed on 3 September 2025). Among these genes, 17 members exhibited distinct expression patterns in leaves and roots under various treatments, including GA, ABA, JA, salt stress, or drought stress. However, *BnaCAMTA3.4* was insensitive to both hormones and stress. In leaves, *BnaCAMTA1.1* and *BnaCAMTA1.2* showed similar downregulation under GA and ABA treatments, but they exhibited different responses to JA. This partial overlap in expression dynamics suggests that their functions may be partially conserved, although further function validation is needed to confirm this. Furthermore, *BnaCAMTA3.3* was significantly upregulated in leaves following JA treatment. The expression of *BnaCAMTA4.2* showed a sharp decrease in root after 1 h of ABA treatment, representing the most pronounced induction observed. Similarly, *BnaCAMTA4.6* exhibited a strong and sustained upregulation in roots after 0.5 h of JA treatment. Notably, *BnaCAMTA1.1* was simultaneously and strongly induced by both GA and ABA, which have antagonistic functions. This convergent response was observed in the roots after 0.5 h of treatment. Under all three hormone treatments, the most intense responses occurred in the roots, indicating that roots serve as a key organ for hormone signal perception and transduction (Figure 7). Collectively, all the results indicated that *BnaCAMTA* genes display diverse expression patterns in response to various phytohormones and stresses, with most genes being more responsive in roots than in leaves. These findings provide valuable insights into the roles of *BnaCAMTA* genes in phytohormone and stress signaling pathways.

Under salt stress, *BnaCAMTA1.1* exhibited early, rapid responses in leaves at 0.25–3 h after the treatment. In contrast, all members in Group II, such as *BnaCAMTA2.1* and *BnaCAMTA2.2*, exhibited strong late-stage responses, underscoring the high conservation of gene function and regulatory timing within this group. Twelve hours after treatment, the expression of multiple genes in leaves peaked. However, the most sensitive phase of gene expression in roots occurred earlier (0.5–1 h). Notably, *BnaCAMTA6.2*, *BnaCAMTA4.4*, and *BnaCAMTA3.3* responded most rapidly and with the greatest magnitude, suggesting their potential roles as key regulators of salt stress response in roots.

**Figure 8:**
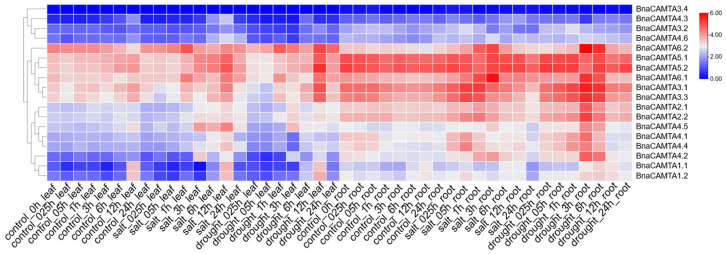
Heatmap analysis of *BnaCAMTA* expression profiles in leaves and roots of ZS11 under salt and drought treatment. The relative expression of *BnaCAMTAs* is represented as Log_2_ (FPKM value + 1).

Under drought stress, a majority of genes in leaves showed peak expression levels at 12 h after treatment, particularly *BnaCAMTA6.2* and *BnaCAMTA5.2*, which were strongly induced. However, *BnaCAMTA1.1* was strongly upregulated as early as 0.25 h, and several genes reached maximum expression around 3 h. In roots, most genes reached their peak expression at 3 h after drought treatment. Interestingly, *BnaCAMTA5.1* and *BnaCAMTA5.2* exhibited sustained high expression levels beginning at 0.25 h and remained stably elevated throughout the treatment. Consistently, under both salt and drought stresses, the expression peaks of most *BnaCAMTA* genes occurred earlier in roots than in leaves, reinforcing the role of roots as the primary sensors of environmental abiotic stress (Figure 8).

### 2.10. Expression Patterns and Functional Analysis of BnaCAMTAs in Response to Salt Stress and GA Treatment

**Figure 9:**
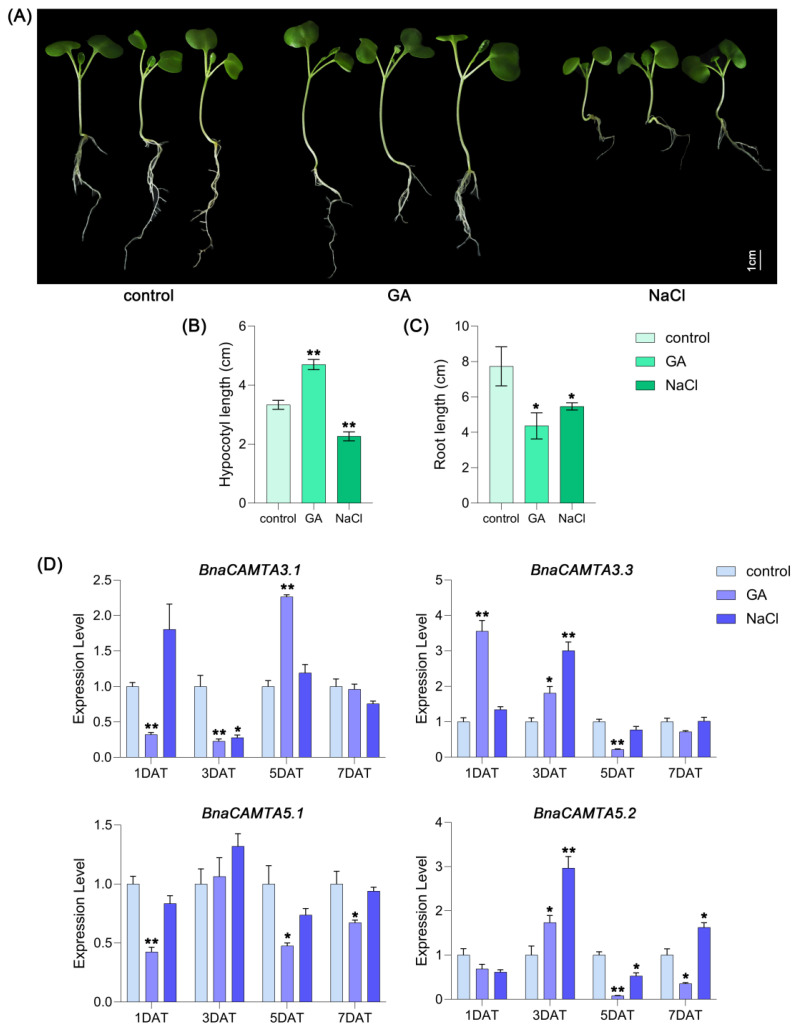
Effects of GA and salt stress on phenotype and *BnaCAMTA* expression levels in *B. napus* seedlings. (**A**) Growth of ZS11 seedlings after 7 days in Hoagland nutrient solution with 150 mM NaCl or 10 µM GA. Bar = 1 cm. (**B**) Effect of NaCl or GA on hypocotyl length of ZS11 seedlings. (**C**) Effect of NaCl or GA on root length of ZS11 seedlings. (**D**) The relative expression level of *BnaCAMTA3.1*, *BnaCAMTA3.3*, *BnaCAMTA5.1*, and *BnaCAMTA5.2* in ZS11 seedlings under salt or GA treatment at the indicated time points (days after treatment, DAT). Error bars represent the standard deviation (SD) of three biological replicates. Statistically significant differences were analyzed by Student’s *t*-test. * *p* < 0.05; ** *p* < 0.01.

After GA and NaCl treatments, the phenotypic responses of *B. napus* seedlings showed significant differences: GA promoted hypocotyl elongation but shortened root length, while NaCl inhibited both hypocotyl elongation and root length (Figure 9A–C). Based on their distinct and antagonistic expression patterns in response to GA treatment and salt stress (Figure 7 and Figure 8), as well as the enrichment of both hormone- and stress-responsive *cis*-elements in their promoters (Figure 6), we specifically selected *BnaCAMTA3.1*, *BnaCAMTA3.3*, *BnaCAMTA5.1*, and *BnaCAMTA5.2* among the differentially expressed *BnaCAMTAs* for further functional analysis. RT-qPCR analysis revealed distinct temporal expression dynamics for these genes under different treatments. Under GA treatment, *BnaCAMTA3.1* expression was significantly suppressed at 1 and 3 days after treatment, exhibited marked induction at 5 days, and subsequently returned to a basal level by 7 days—a pattern that diverged substantially from its homolog *BnaCAMTA3.2.* In contrast, *BnaCAMTA5.1* was generally downregulated throughout the treatment period except for transient elevation at 3 days, whereas *BnaCAMTA5.2* showed transient induction at 3 days followed by sustained downregulation. Under salt stress, *BnaCAMTA3.1* was specifically downregulated at 3 days, while *BnaCAMTA3.3* exhibited concomitant transient upregulation. *BnaCAMTA5.1* expression remained largely unchanged, whereas *BnaCAMTA5.2* displayed a dynamic profile characterized by significant induction at 3 days, followed by a decline and subsequent recovery (Figure 9D). These expression patterns indicate functional divergence between homologous gene pairs: *BnaCAMTA3.1* and *BnaCAMTA3.3* appear to participate primarily in early signal events, whereas the protracted expression dynamics of *BnaCAMTA5.2* correlate more strongly with phenotypic adaptation. Collectively, these findings implicate *BnaCAMTA5.2* as a pivotal regulatory node mediating crosstalk between salt stress and GA signaling pathways, likely playing a crucial role in coordinating root growth with stress adaptation in *B. napus*, thereby underscoring its significance in plant adaptive response.

### 2.11. Comprehensive Analysis of Potential miRNAs Targeting BnaCAMTAs

Numerous studies have highlighted the crucial roles of miRNAs in regulating plant growth and development [42,43]. Our analysis revealed that 17 *BnaCAMTA* genes were predicted to be targeted by 122 miRNAs (Table S3). Notably, *BnaCAMTA4.2* was an exception, with no predicted miRNA targets. The candidate miRNAs were classified into 17 families. Among them, miR169 was found to potentially regulate the largest number of *BnaCAMTAs* (22), while the remaining miRNAs targeted 1 to 20 *BnaCAMTAs* genes each. Except for *BnaCAMTA5.2* and *BnaCAMTA4.2*, each *BnaCAMTA* gene was targeted by more than two miRNAs, with *BnaCAMTA3.1* being the most prominent, targeted by 18 miRNAs. Many of the predicted miRNAs are known to be stress-responsive, such as miR169, which has been extensively documented in drought stress responses [44,45]. Intriguingly, the expression profiles revealed that several *BnaCAMTA* genes, which are predicted to be targeted by stress-responsive miRNAs (e.g., *BnaCAMTA3.1* by miR169), exhibited delayed or attenuated upregulation under drought and salt stress conditions (Figure 8). This observation is consistent with the potential repressive role of these miRNAs in regulating gene expression under stress. Given the function of CAMTAs in coordinating abiotic stress responses, these findings suggest that miRNAs may function as key post-transcriptional regulators, fine-tuning the expression of these master regulatory genes.

## 3. Discussion

The CAMTA gene family plays a pivotal role in calcium-mediated signal transduction and significantly regulates responses to hormones and stresses [33,37]. Although extensive research has been conducted on model species such as *Arabidopsis thaliana* [28], comprehensive genome-wide analyses of the CAMTA gene family in the economically important *Brassica* U-triangle species have been relatively limited. In this study, we identified 64 CAMTA genes from six *Brassica* species and systematically investigated their phylogenetic relationships, gene structures, conserved domains, chromosomal distribution, and expression patterns under hormonal and abiotic stress conditions, as well as the potential miRNA prediction.

Gene duplication and loss are widespread phenomena in plants and serve as important evolutionary drivers of phenotypic diversification and environmental adaptation [46]. Polyploids, particularly allopolyploids, integrate multiple divergent yet compatible progenitor genomes, often triggering large-scale gene loss during evolution [47]. Our analysis of the CAMTA gene family provides a concrete example of this pattern: phylogenetic analysis revealed that *Brassica* CAMTAs are classified into four major groups, consistent with the classification in *A. thaliana*, indicating a high degree of evolutionary conservation. However, the number of CAMTA family members did not simply increase linearly from *A. thaliana* to *Brassica* species. Similarly, in allotetraploid species, the CAMTA gene repertoire was reshaped in a non-additive manner, deviating from the simple sum of its diploid progenitors. For instance, in *B. napus* (18 genes), the count not only exceeds the simple sum of its progenitors (*B. rapa*: 9 + *B. oleracea*: 5 = 14) but also exhibits lineage-specific fates. A case in point is the *CAMTA2* lineage: absent in *B. oleracea*, it was introduced into *B. napus* via the *B. rapa* subgenome and subsequently expanded, contributing to the net increase in gene number. This exemplifies how polyploidization triggers a dynamic process of gene loss, retention, and new duplication, with the final gene content likely influenced by gene dosage balance and functional constraints. This phenomenon is consistent with patterns reported in the PYL gene family in *Triticum aestivum* [48], the MYB gene family in *Solanum tuberosum* [49], and the CAD and GGCT gene families in *B. napus* [50,51]. Further analysis indicated that the expansion of the CAMTA gene family is primarily attributed to whole-genome duplication and segmental duplication, supported by the highly similar exon–intron structures and conserved motif compositions within the same group. Notably, the CaMBD domain, essential for calcium-dependent calmodulin binding, is highly conserved across all species, underscoring the stability of its core function.

The similarity in gene structure and motif composition among members within each subgroup further reflects the coexistence of structural conservation and dynamic changes during the evolution of this gene family. Chromosomal distribution further revealed that retained genes often resided in collinear blocks with high synteny conservation (e.g., on A09), suggesting that genomic context influences post-polyploidization gene fate. Furthermore, Ka/Ks analysis indicated that most orthologous pairs have undergone strong purifying selection (Ka/Ks < 0.3). However, a subset of pairs exhibited higher values (0.5–0.78, Table S2), potentially indicating periods of relaxed selection or incipient functional divergence in specific lineages. Collectively, these findings illustrate a multifaceted evolutionary trajectory: while core CAMTA functions are largely conserved under purifying selection, polyploidization triggered lineage-specific gene loss and retention, with genomic synteny and phylogenetic membership influencing which genes were preserved in the allopolyploid species.

This study revealed that *BnaCAMTA* genes exhibit significant temporal expression dynamics in response to stress. For instance, *BnaCAMTA3.1* was suppressed during the early stage of gibberellic acid treatment but activated at later stages, whereas *BnaCAMTA5.2* showed an initial induction followed by a subsequent decline under salt stress. Such dynamic expression patterns reflect the widespread time-dependent regulatory characteristics of plant genes in responding to environmental signals, likely corresponding to distinct physiological phases such as stress perception, resource reallocation, and adaptive reconstruction [39]. These findings indicate that CAMTA transcription factors play a temporally regulated role in coordinating plant growth and stress adaptation, providing a new temporal perspective for understanding plant adaptive mechanisms in dynamic environments.

Beyond elucidating evolutionary patterns, our analysis provided valuable insights into potential regulatory diversification within this gene family. Notably, the gene structure analysis revealed that specific members, including *BraCAMTA1* and *BjuCAMTA4.3*, exhibit extended UTRs or introns. Verification against genomic and transcriptomic data confirmed the integrity of their coding sequences. While the core protein architecture remains strictly conserved across these genes, the structural variations observed in non-coding regions may introduce an additional layer of regulation complexity. These extended regions could influence mRNA stability, subcellular localization, or translational efficiency. Moreover, they may harbor *cis*-elements or miRNA binding sites that fine-tune gene expression in response to specific developmental or environmental cues. Consequently, these structural features may contribute to the functional plasticity observed within this gene family.

Analysis of promoter regions revealed an abundance of *cis*-elements related to ABA, JA, and GA signaling pathways, as well as light signaling and abiotic stress responses. This suggests that CAMTA genes are regulated by complex mechanisms and may function in signal crosstalk. This finding aligns with the results from other species: *AtCAMTA1* in *Arabidopsis* participates in drought stress response [30]; *OsCAMTA4/5/6* in *Oryza sativa* enhance cold tolerance by regulating reactive oxygen species homeostasis [37]; and *PvCAMTA1/2/3/4/5/8* in *Phaseolus vulgaris* are significantly upregulated under salt stress [38]. Compared to their diploid progenitors, CAMTAs in the allopolyploids, such as *B. napus*, exhibit greater diversity of *cis*-elements, implying that genome duplication may have facilitated regulatory innovation and enhanced adaptive plasticity. Expression profiling showed that *BnaCAMTA* genes display tissue-specific expression, predominantly in roots and siliques. Under phytohormone (GA, ABA, JA) and abiotic stress (salt, drought) treatments, the response of *BnaCAMTA* genes was more rapid and pronounced in roots than that in other organs or tissues, confirming that roots are primary sensors of environmental signals.

The observed diversity in spatiotemporal and stress-responsive expression among *BnaCAMTAs* indicates that functional specialization has occurred within this gene family. This specialization likely operates through the evolution of regulatory elements—such as the diverse *cis*-acting motifs and potential miRNA targeting sites identified—while the conserved protein domain architecture is maintained to execute the core biochemical function of calcium-mediated transcriptional activation.

Consistent with this model of regulatory diversification, our study has revealed that *BnaCAMTA5.2* exhibits antagonistic effects on hypocotyl and root growth under GA or salt stress conditions. Specifically, GA treatment promotes hypocotyl elongation while simultaneously suppressing root growth, whereas salt stress exerts a suppressive influence on both hypocotyl and root development. The contrasting expression patterns of this gene in response to these treatments suggest that it may function as a molecular switch, orchestrating a balance between growth promotion and stress adaptation. This finding provides insights into the mechanisms by which CAMTAs integrate multiple signaling pathways to enhance plant fitness under fluctuating environments.

MicroRNAs (miRNAs) are crucial regulators of gene expression and play key roles in plant growth, development, and stress responses [42]. The present study identified several stress-associated miRNAs that potentially target *BnaCAMTAs*, especially the drought-responsive miR169 [44], suggesting widespread miRNA involvement in the post-transcriptional regulation of CAMTA genes. Furthermore, regulation at the protein level is also evident, as exemplified by a recent study demonstrating that CAMTA can interact with diverse signaling components, including transcription factors and phosphatases, forming regulatory modules [52]. Further research should be performed to validate these potential regulatory interactions employing degradome sequencing or dual-luciferase reporter assays. Elucidating these multi-layered post-transcriptional and post-translational regulatory interactions could ultimately reveal novel mechanisms for fine-tuning stress responses and contribute to improving stress resistance in *B. napus* through molecular breeding.

## 4. Materials and Methods

### 4.1. Identification and Annotation of CAMTAs

The genome information on *AtCAMTAs* was obtained from the TAIR database (https://www.arabidopsis.org (accessed on 16 September 2025)) [53], and the genome data of the U-triangle (*B. carinata*, *B. oleracea*, *B. juncea*, *B. napus*, *B. nigra*, and *B. rapa*) were downloaded from the *Brassica napus* multi-omics information resource database (BnIR, https://yanglab.hzau.edu.cn/BnIR [54] (accessed on 31 August 2025)). Six *AtCAMTAs* (*AT5G09410.3*, *AT5G64220.1*, *AT2G22300.1*, *AT1G67310.1*, *AT4G16150.1*, and *AT3G16940.1*) were used as seed queries to predict gene members in *Brassica* species. The NCBI Blastp program (https://blast.ncbi.nlm.nih.gov/Blast.cgi?PROGRAM=blastp&PAGE_TYPE=BlastSearch&LINK_LOC=blasthome (accessed on 31 August 2025)) and TBtools-BLAST (v2.376) were used to identify the CAMTAs with default parameters [55,56]. To confirm whether the candidate CAMTA members truly belong to the CAMTA family, the National Center of Biotechnology Information’s (https://www.ncbi.nlm.nih.gov/Structure/cdd (accessed on 31 August 2025)) conservative structure domain database (CDD) was used [57]. TBtools-Protein parameter Calc was utilized to predict the length (number of amino acid residues), molecular weight (MW, in kDa), isoelectric point (pI), and instability index of each CAMTA protein [55]. Plant Cell-PLoc 2.0-mPLoc (http://www.csbio.sjtu.edu.cn/bioinf/plant-multi/ (accessed on 31 August 2025)) was used to predict the subcellular locations of proteins [58].

### 4.2. Phylogenetic Analysis of CAMTA Gene Family Members

A phylogenetic tree was constructed based on 70 CAMTA protein sequences using the MEGA 12 software [59]. The sequences were aligned with ClustalW under default settings, and the tree was constructed employing the maximum-likelihood (ML) method. The best-fit model, JTT with Freqs. (+F) and Gamma Distributed With Invariant Sites (G+I), was applied. For graphical refinement, the phylogenetic tree was processed using EvolView. (http://www.evolgenius.info/evolview/ [60] (accessed on 31 August 2025)).

### 4.3. Sequence Analysis of CAMTA Gene Family Members

To gain CaMBD sequences in *Brassica* U-triangle species, the MEGA 12 software was used [59]. CaMBD sequences in *Arabidopsis* were obtained from previous reports [31]. NLStradamus (http://www.moseslab.csb.utoronto.ca/NLStradamus/ (accessed on 31 August 2025)) was used to predict the position and sequence of NLS [61]. Other domain information was obtained from the National Center of Biotechnology Information’s (https://www.ncbi.nlm.nih.gov/Structure/cdd (accessed on 31 August 2025)) conservative structure domain database (CDD) [57]. The domain structures of CAMTAs were drawn using Domain Graph software (http://dog.biocuckoo.org/ (accessed on 31 August 2025)) [62]. The sequence logo of the CaMBD domain was generated by TBtools-SeqLogo [55].

### 4.4. Conserved Protein Motifs and Gene Structures of CAMTA Family Members

The online website Multiple Expectation Maximization for Motif Elucidation (MEME, https://meme-suite.org/meme/doc/meme.html (accessed on 1 September 2025)) [63] was used to identify and analyze the conserved motifs of CAMTA family members, with a maximum number of motifs set to 10. The motifs and gene structure were visualized using TBtools-Gene Structure View (Advanced) [55].

### 4.5. Chromosomal Localization and Collinearity Analysis of CAMTA Genes

The chromosomal location information of CAMTA family genes was extracted using the genome sequence annotation of *Brassica* species. The CAMTA family genes were mapped to their corresponding chromosomes using MG2C_v2.1 (http://mg2c.iask.in/mg2c_v2.1/ (accessed on 1 September 2025)) to visualize [64]. To better identify the collinearity relationship, TBtools-One Step MCScanX and TBtools-Amazing Super Circos [55] were utilized to analyze the homologous relationships among *CAMTA* genes. Then, TBtools-Simple *Ka*/*Ks* Calculator was employed to calculate the nonsynonymous substitution rates (*Ka*), synonymous substitution rates (*Ks*), and *Ka*/*Ks* ratios of homologous genes to estimate the selection pressure during evolution [55].

### 4.6. Cis-Element Analysis in the Promoter Regions of CAMTA Genes

TBtools-Gtf/GFF3 was used to obtain the 2000 bp upstream sequences of each CAMTA gene as the promoter region [55]. The *cis*-elements were analyzed using the Plant CARE website (http://bioinformatics.psb.ugent.be/webtools/plantcare/html/ (accessed on 3 September 2025)) [65]. Subsequently, the results were categorized and visualized through TBtools-Simple BioSequence Viewer [55].

### 4.7. Expression Profiling of BnaCAMTA Family Genes

RNA-seq data were obtained from BnIR (https://yanglab.hzau.edu.cn/BnIR/expression_zs11 (accessed on 3 September 2025)) to investigate the tissue-specific expression patterns of *BnaCAMTAs*, as well as their roles in response to hormones and abiotic stresses [54]. The relative expression levels of the genes were normalized using the Log_2_ (FPKM value + 1) method and were generated with TBtools to illustrate the expression profiles of *BnaCAMTA* genes [55].

### 4.8. Plant Materials and Treatment

Zhongshuang11 (ZS11) is a leading cultivar of *Brassica napus* in China. It possesses excellent agronomic traits such as high oil content and strong stress resistance. Moreover, its genome background is well-defined, and it has been widely used in previous research, making it a representative and reliable choice for experimental materials. All plant materials were grown in a growth chamber under a 16 h light/8 h dark photoperiod at 22 °C, with a light intensity of 100 μmol/m^2^/s. ZS11 seeds were placed in a Petri dish with wet filter paper and germinated for 3 days. Well-germinated seedlings were selected and placed on the floating platforms submerged in containers filled with Hoagland nutrient solution, containing 150 mM NaCl or 10 µM GA, where they were grown for 7 days [66,67]. Samples were then collected and immediately frozen in liquid nitrogen and stored for subsequent analysis.

### 4.9. RT-qPCR Analysis of BnaCAMTA5.2

The primers required for RT-qPCR analysis were designed using the website BrassicaEDB-qPrimer (https://brassicaedb.com/#/tools/qprimer (accessed on 6 December 2025)) [68] and are listed in Table S5. Total RNA was extracted using the EZ-10 DNAaway RNA Mini-Preps Kit (Sangon Biotech Co., Ltd., Shanghai, China). The cDNA was synthesized using the ExonScript RT Mix (with dsDNase) kit (Baoguang, Chongqing, China). RT-qPCR was performed using the Bio-Rad CFX96 Real Time System (Bio-Rad Laboratories, Hercules, CA, USA). Each experiment was conducted with three replicates. Data normalization was performed using the 2^−ΔΔCT^ calculation method [69] using *BnaActin7* as the internal standard.

### 4.10. MiRNA–Target Analysis

The potential regulatory miRNAs of *BnaCAMTAs* were predicted using the psRNATarget website (https://www.zhaolab.org/psRNATarget/analysis?function=1 (accessed on 6 September 2025)) [70].

## 5. Conclusions

The present study elucidates the evolutionary patterns and functional diversity of the CAMTA gene family within *Brassica* U-triangle species. Our findings indicate that the expansion of the CAMTA gene family is predominantly driven by polyploidization events, and these genes have been subject to strong purifying selection during evolution. Despite this, the core functional domains and gene structures exhibit high conservation across different groups. Further analysis of regulatory elements and expression profiles suggests that CAMTA genes may integrate multiple hormonal and abiotic stress signals. Notably, *BnaCAMTA5.2* was identified as a key candidate gene that coordinates salt stress and GA signaling in *B. napus*. Furthermore, the prediction of stress-associated miRNAs targeting *BnaCAMTAs* not only provides valuable resources for future research but also proposes a novel layer of post-transcriptional regulation that could be exploited for enhancing stress resistance in *Brassica* crops.

## Data Availability

All other datasets supporting the results of this article are included within the article and Supplementary Materials.

## Supplementary Materials

The following supporting information can be downloaded at https://www.mdpi.com/article/10.3390/plants15030480/s1, Figure S1. Heatmap of the expression patterns of *BnaCAMTAs* across different organs from ZS11 at different developmental stages; Table S1: Characteristics of CAMTAs from *Arabidopsis* and the *Brassica* U-triangle species; Table S2: The *Ka/Ks* ratio between the orthologous gene pairs in *A. thaliana* and *Brassica* U-triangle species; Table S3: Predicted miRNA–target pairs between *B. napus* miRNAs and CAMTA genes; Table S4: Summary of *cis*-elements identified in the promoter regions of CAMTA genes across *A. thaliana* and U-triangle species; Table S5: Primers used for RT-qPCR analysis.
