# Supplementary material for: Genome-Wide Identification and Characterization of the Calmodulin-Binding Transcription Activators (CAMTA) Gene Family in Brassica U-Triangle Species and Its Potential Role in Response to Phytohormones and Abiotic Stresses
Source: Plants (Basel). 2026 Feb 3;15(3):480. doi: 10.3390/plants15030480 (PMC12899841; doi:10.3390/plants15030480)
Supplement: Supplementary file 1 [file plants-15-00480-s001.zip › Figure S1.pdf]

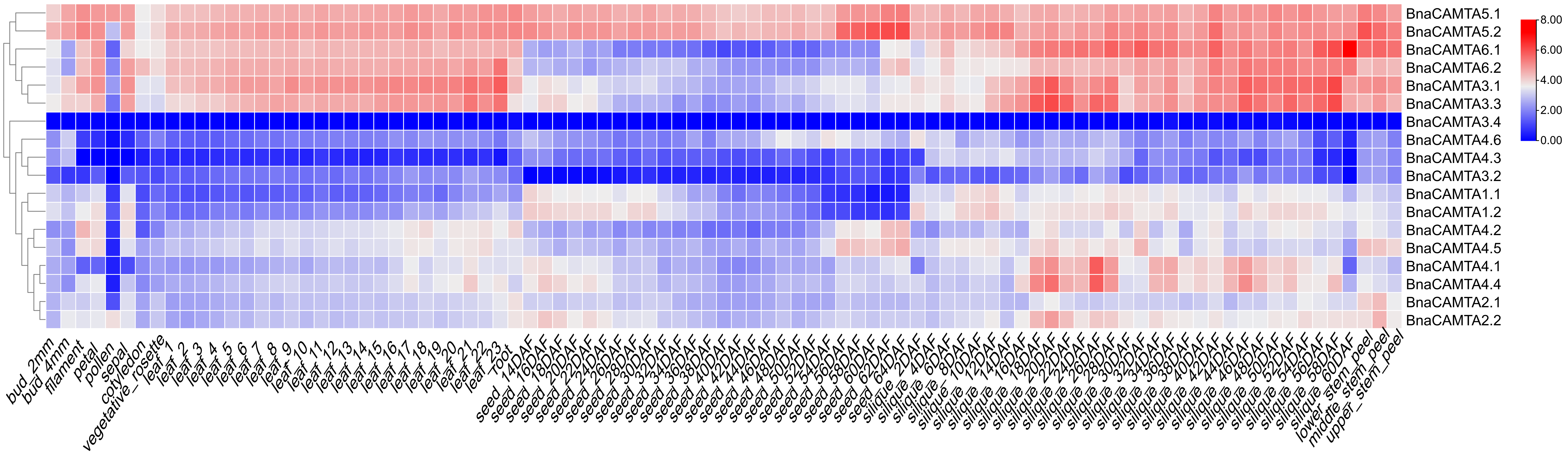

Figure 7. Heatmap of the expression patterns of BnaCAMTAs across different organs from ZS11 at different developmental stages. The expression profiles of each BnaCAMTAs gene are based on log2-transformed values (FPKM value +1). DAF denotes days after flowering.
